# Chronic Mollescence of the Spinal Cord

**Published:** 1830-04-01

**Authors:** 


### I. CHRONIC RAMOLLISSEMENT OF THE SPINAL CORD.

One case of this kind which fell under his observation, and one only, is related by Dr. Abercrombie in the last edition of his work upon the Brain and Spinal Cord. The disease, says the Doctor, may go on for a considerable time, sometimes for years, before it is fatal. There is generally some uneasiness in the back, with paralytic symptoms, beginning in a slight degree in a part of a limb and advancing very gradually to confirmed palsy. Most commonly the lower extremities only are affected, in some cases the arms only, in others all the limbs. There is sometimes permanent contraction of the affected limbs, sometimes spasmodic affections of them; and in this manner the disease may go on for many years, " and at last be fatal by ramollissement." The last part of this history seems rather superfluous, for if the disease *be* ramollissement how can it prove fatal by *becoming* such? The varying nature of the symptoms enumerated is sufficient to convince any one, if any one were previously ignorant of the fact, that the only diagnostician of chronic ramollissement of the spinal cord, is the scalpel of the anatomist, post mortem. We suspect indeed that the cases that have fallen under the Doctor's notice have not been very numerous, but *n'importe;* we will give the particulars of the sole illustration from his own practice with which we are furnished.

*Case.* A gentleman, aged 42, in

October 1827, began to be affected with pain in the lower part of the back, stretching round the abdomen, and frequently shooting into the groins. After a short time this was succeeded by coldness and numbness of his feet, which gradually extended upwards with diminished power of motion, until, after several weeks, it terminated in perfect loss of motion of both lower extremities, with retention of urine. There was pain in some parts of the affected limbs, and in others a painful sensation of cold. This perfect loss of power continued five or six weeks, when, after a great deal of treatment by cupping, blistering, &c. he recovered a slight degree of motion, but no power of the bladder. He then began to be affected with spasms of the muscles of the back and abdomen, with a very uneasy sensation of tightness across the abdomen, and at times across the lower part of the thorax. The spasms occasionally assumed the characters of opisthotonos, and at one time he had almost incessant hiccup, which continued in a most violent degree for several days. After the employment of various antispasmodics, this subsided under the use of musk. During the course of these symptoms, he frequently complained of pain in various parts of the spine, at first in the lower part, and afterwards higher up; and the feeling of numbness extended gradually upwards, till it reached nearly the upper part of the dorsal region, and was felt in a very considerable degree along the sides of the thorax.

After this he became liable to feverish attacks at night, terminating in the morning by very profuse perspiration, but this was strictly confined to the parts which were not palsied, and there never was the smallest moisture on the lower extremities. He had also, in the upper extremities, a frequent feeling of intense heat, while the lower continued cold and benumbed. During this time a considerable, but very imperfect, degree of motion continued in the lower extremities, but the bladder continued entirely paralytic.

In April, 1828, he went to the country, and at this time he had such a degree of motion as to walk a little on a smooth garden walk, leaning on two persons, or supported by crutches. But soon after this he began to complain of pain in the head. It occurred in irregular paroxysms, and was often referred to a small defined spot, on various parts, especially behind the ear, and sometimes to the tip of the ear. This pain seemed to abate under the use of arsenic; but soon returned, and became more fixed and permanent, and the palsy of the limbs again increased. After an absence of about two months, he returned to town in the beginning of July. At this time the headach was severe, and the power of the limbs so much impaired, that he was entirely confined to bed. In a few days after his return, the right arm became paralytic, and his speech considerably impaired. After a day or two these symptoms rather subsided, but in the following night he became comatose, and died in the afternoon. There never was complete loss of sensation of the affected limbs; he had only complained of it occasionally at particular spots, and of a general feeling of numbness and coldness.

*Sectio Cadaveris.* Some scales of bone loosely attached to the inner surface of the theca vertebralis — whole cord of a pale rose colour, and in a state of complete ramollissement throughout, being entirely diffluent in every part. A slight degree of softening on the anterior part of the medulla oblongata; a little softening also on the tuber annulare, apparently involving the origin of the fifth nerve (on which side not stated.) Beyond this, the ramollissement became again more decided, extending along the crura cerebri and cerebelli, and considerably into the substance of the brain at the part adjoining the crura. Brain in other respects healthy—no effusion in ventricles.

Dr. Abercrombie justly remarks that in such a case as this it is difficult to trace the precise nature and progress of the affection of the cord. The Doctor, as our readers well know, maintains that the ramollissement of the brain originates in a low chronic kind of inflammation, and evidently argues by implication that it is so with the softening of the spinal cord. It is difficult to imagine how any morbid process can go on without a local action of the vessels, which may be construed by those who like it into inflammation; and if, as some clever men argue, mere growth be inflammation, why we suppose that there is no disputing the matter. But this after all is only begging the question, and such "low chronic" inflammatory action may exist or may not, is, strictly speaking, no inflammation at all; and, practically speaking, is hardly to be treated as such. Several interesting cases are cited by Dr. Abercrombie from Olivia, Andral, and others, but for these we must refer our readers to the work itself.

II. Caries of the Odontoid Process—Fungous Tumour of the Dura Mater.

*Case.* A gentleman, aged 22, of a scrofulous habit; in the early part of his life had suffered amputation on account of a disease of the knee, and afterwards was liable to pectoral complaints with hæmoptysis. In the beginning of the year 1828, he began to complain of pain and stiffness of the neck, referred chiefly to the left side of it, and much increased by the motion of the head. The pain sometimes extended into the larynx, and backwards towards the scapula. After considerable relief from repeated blistering, &c. the symptoms returned, accompanied by loss of appetite, frequent pulse and night perspirations; and soon after this he became affected with difficult deglutition, some dyspnœa and hoarseness. There was now also severe fixed pain referred to the back of the head, and much increased by the motion of the parts; so that he was obliged to support his head with both his hands when he had occasion to make any change of his posture. He was next affected with paralysis of the tongue and the upper eyelid of the left side. On 16th January, 1829, he was seized with paralysis of the left arm, and two days after, the right was affected in the same manner. He had then great pain and difficulty in passing urine, with obstinacy of the bowels, which nothing could overcome. On the 29th, the lower extremities became paralytic, and he died on the 31st, having suffered greatly on the day on which he died, from difficult breathing.

*Sectio Cadaveris.* External parts of neck, pharynx, &c. healthy—no disease of vertebræ discoverable externally. Brain and cerebellum rather vascular, but otherwise healthy. Within the foramen magnum, and attached to the inner surface of the dura mater at its anterior and lateral parts, was a spongy tumour of a greyish-yellow colour, which, when cut into, presented a variegated structure resembling fungus hæmatodes. The processus dentatus rough and carious on its surface, and so much elongated as to project half an inch into the cavity of the cranium; its ligaments partially destroyed so as evidently to allow it to encroach upon the area of the spinal canal and to compress the cord. The spinal cord at the upper part flattened, but not materially altered in its texture.

It is a consolatory thing to know, that these cases of disease of the upper cervical vertebræ are frequently of a more favourable, or rather less fatal, nature, than they appear to be. We have witnessed several instances of recovery, where the symptoms demonstrated the existence of considerable mischief about the second and third vertebræ of the neck, and in one case particularly where an eminent surgeon formed an extremely unfavourable prognosis. Perfect repose, counter-irritation by blisters and especially by caustic issues, the moxa or stimulating liniments when only stiffness remains, and the employment of constitutional remedies according to the general indications, are the means which we have seen of the greatest service. It is in such patients as these, when the frame is delicate and scrofulous, the hectic slight, and the pulse inclined to be feeble, that bark and the liquor potassæ are often of much benefit. They require, however, to be used with discrimination.

